# Abstract of Clinical Lecture in the Long Island Hospital on the Diagnosis of Syphilis

**Published:** 1916-02

**Authors:** Henry H. Morton

**Affiliations:** Clinical Professor of Genitourinary Diseases in the Long Island Hospital; Genitourinary Surgeon to Long Island College and Kings County Hospitals and the Polhemus Memorial Clinic, Etc., Brooklyn, N. Y.


### Diagnosis of Syphilis by Laboratory Methods.

BY HENRY H. MORTON, M. D., CLINICAL PROFESSOR OF GENITO-URINARY DISEASES IN THE LONG ISLAND HOSPITAL; GENITO-URINARY SURGEON TO LONG ISLAND COLLEGE AND KINGS COUNTY HOSPITALS AND THE POLHEMUS MEMORIAL CLINIC, ETC., BROOKLYN, N. Y.

We have four ways of making an examination for the purpose of making a diagnosis of syphilis:

1. The demonstration of the spirocheta pallida in the suspected lesion.
2. The Wasserman reaction in the blood.
3. The cutaneous or Luetin reaction of Noguchi.
4. The findings in the spinal fluid.

Clinical experience must supplement laboratory diagnosis, for sometimes laboratory reports are misleading.

For the demonstration of the spirocheta the "Dark Field" is the best method, as the living spirocheta may be seen.

The India ink and Giemsa stains are also extensively used for the demonstration of spirocheta in smears, while the Levaditi stain is used to demonstrate the spirocheta in tissues.

Th Wasserman reaction is depended upon for the diagnosis of syphilis today. The old method is the best, as short cuts are unreliable.

A positive reaction appears six to eight weeks after infection. One hundred per cent of secondary syphilitics untreated give a positive reaction.

In some cases, in spite of good treatment, it is impossible to change the reaction, if, however, it is reduced but is not negative it shows the need of more treatment.

In suspected cases where the Wasserman is negative a small dose of salvarsan or mercury will often cause the reaction to be positive in twenty-four to forty-eight hours. This is called a provocative Wasserman.

The Luetin reaction was devised by Noguchi in 1911. A mixed culture of killed spirochetae are injected beneath the skin. A positive reaction appears as a papule or pusule in three to five days. Nichols and Craig found that the positive reaction appeared more consistently in latent cases.

The findings in the spinal fluid are useful in diagnosing cerebro-spinal syphilis. There is very little danger in lumbar puncture if properly done.

In examining the spinal fluid we estimate the pressure, make a cell count, estimate the globulin and do a Wasserman.

Up to five cells per cm. is normal. Five to ten is doubtful, and over ten is pathological. Increased cell count shows irritation along the cord.

Globulin is not normally found in spinal fluid, and its presence shows irritation. It is present in 80 to 100 per cent of syphilitic nervous disease.

In syphilitic nervous disease the Wasserman reaction is usually present. In Tabes about 60 to 70 per cent positive. In Paresis 100 per cent positive.

All laboratory reports must come from reliable laboratories, else reports will be misleading and then clinical experience must determine the diagnosis.

### Blood and Melancholia.

The practice of blood-letting common in the eighteenth century, especially in England, but which practically disappeared both in that country and in America in the first half of the nineteenth century, has within a few years regained some of the favor if none of the fury that one characterized this sanguinary form of the regular practice. An eminent English surgeon, in the *London Lancet* for September, proffers "a simple little remedy for melancholia." He would let out some ounces of the bad blood which is doing all the mischief and thus relieve the pressure on the brain. He says that the prompt use of the scalpel may prevent a suicide or incarceration in a madhouse. He then tells of a melancholy young Englishman whom he relieved of about twenty ounces of blood. This saved him from suicide, restored him to mental health, and now he has enlisted and gone to the front.

